# Total Oxidant and Antioxidant Status in Prepubertal Children with Obesity

**DOI:** 10.1155/2017/5621989

**Published:** 2017-08-20

**Authors:** Grażyna Rowicka, Hanna Dyląg, Jadwiga Ambroszkiewicz, Agnieszka Riahi, Halina Weker, Magdalena Chełchowska

**Affiliations:** ^1^Nutrition Department, Institute of Mother and Child, Warsaw, Poland; ^2^Screening Department, Institute of Mother and Child, Warsaw, Poland

## Abstract

**Aims:**

Obesity is accompanied by the formation of oxygen free radicals, whose intensified activity without effective defense mechanisms can lead to oxidative stress and related complications. We evaluated the presence of oxidative stress in obese prepubertal children.

**Methods:**

The study included 83 healthy children aged 2–10 years (62 with obesity and 21 nonobese controls). Total oxidant capacity (TOC), total antioxidant capacity (TAC), oxidized low-density lipoprotein (ox-LDL), lipid parameters, glucose, and C-reactive protein (CRP) were measured in serum. Oxidative stress index (OSI) was calculated.

**Results:**

Serum TOC concentration was significantly higher (*p* < 0.05) and TAC concentration was lower (*p* < 0.05) in obese children. OSI was higher (*p* < 0.01) in obese subjects compared with controls. CRP levels were normal in all children, but median CRP value was higher (*p* < 0.01) and HDL cholesterol levels were lower (*p* < 0.05) in the obese group. We found a significant negative correlation between TAC and ox-LDL concentrations (*r* = −0.27, *p* < 0.05) in obese children. Furthermore, obesity duration was positively correlated with TOC level (*r* = 0.32, *p* < 0.05) in this group.

**Conclusions:**

Obesity-related oxidative stress already occurs in prepubescence. Early obesity diagnosis and the necessary therapeutic activity implementation is a vital strategy for the prophylaxis of free radical damage and related multiorgan complications.

## 1. Introduction

Over the last few decades, we have observed a steady growth in the frequency of obesity occurrence, not only among adults but also in children and adolescents. The global incidence of childhood obesity varies depending on the country and the year; however, there is an overall increase [1–4]. Studies show that approximately 40% of overweight children will continue to have increased weight during adolescence, and 75–80% of obese adolescents will become obese adults. A child with a high BMI has a high risk of being overweight or obese at 35 years old, and this risk increases with age [5, 6]. The majority of obesity in adulthood has its origins in childhood, which makes obesity a pediatric concern and the period when interventions should be undertaken [4].

Energy imbalances lead to the storage of excess energy in adipocytes, resulting in both hypertrophy and hyperplasia. These processes are associated with abnormalities in adipocyte functioning. Obesity alters the metabolic and endocrine functions of adipose tissue and leads to increased release of hormones, fatty acids, and proinflammatory molecules that contribute to obesity-related complications [7]. Proinflammatory mediators released from adipose tissue cannot only cause direct endothelial damage but also generate excess free radical formation. Such activities are exhibited by, for example, TNF-*α* (tumor necrosis factor alpha), Il-6 (interleukin 6), CRP (C-reactive protein), leptin, and resistin [8].

The formation of reactive oxygen (ROS) and nitric oxide (NOS) species is an intrinsic phenomenon accompanying biochemical changes occurring in the human body, which has developed mechanisms to protect biomolecules from the deleterious effects of free radicals [9]. These include the enzymes superoxide dismutase (SOD), catalase (CAT), and glutathione peroxidase (GPx) and water and lipid-soluble antioxidants, such as glutathione, ascorbate (vitamin C), *α*-tocopherol (vitamin E), and *β*-carotene, and also endogenous antioxidant, for example, albumin, bilirubin, and uric acid [10, 11]. Much evidence indicates that white adipose tissue mitochondria, particularly in people with obesity, are the main site of ROS generation, accompanied by augmented expression of NDPH (nicotinamide adenine dinucleotide phosphate) oxidase and decreased expression of antioxidative enzymes [12].

An imbalance between the formation of ROS in cells and antioxidant defense causes oxidative stress, which is responsible for oxidative damage to lipids, proteins, and nucleic acids, and modifies their structure as well as functioning [13].

Oxidative damage to important cellular structures is one of the factors responsible for the development of obesity-related complications, such as atherosclerosis, hypertension, ischemic heart disease, insulin resistance, and type 2 diabetes [9, 14]. Obesity significantly increases the risk of developing these diseases and early death as a result [5, 15].

Many markers are currently used to assess oxidant and antioxidant status. They include total oxidant capacity (TOC), total antioxidant capacity (TAC), oxidative stress index (OSI), which expresses the TOC/TAC ratio, and oxidized low-density lipoproteins (ox-LDL), which are lipid peroxidation metabolites [16–19]. There are many studies on oxidative stress in adults with obesity, whereas knowledge about the body's reaction to oxidative stress caused by obesity in children, particularly the younger ones, is limited.

The aim of our study was to assess the severity of oxidative processes (TOC, ox-LDL, and OSI) as well as total antioxidant capacity (TAC) in children with obesity aged 2–10 years.

## 2. Materials and Methods

The study was performed in accordance with the Helsinki Declaration for Human Research, and the study protocol was approved by the Ethics Committee of the Institute of Mother and Child in Warsaw, Poland. All parents of the participating children were informed of the study's objectives, and written consent was obtained for blood sample analysis before participation in the study.

### 2.1. Subjects

The study was conducted at the Institute of Mother and Child in Warsaw between January 2014 and June 2016. The study included 83 healthy children aged 2–10 years. Group I (*n* = 62) consisted of children with obesity, wherein the criterion for obesity diagnosis in children up to 5 years old was BMI z-score ≥ 3SD, and in children over 5 years old BMI z-score ≥ 2SD [20]. Group II (*n* = 21) included nonobese children whose BMI z-score was <−1 + 1>. Exclusion criteria included infections of various etiologies and localizations as well as intake of prescription medications and food supplements with antioxidant properties.

### 2.2. Anthropometric Measurements

Height and weight were assessed using a standard stadiometer and electronic scale, respectively. Anthropometric measurements were taken using calibrated instruments. The same team examined all the study participants. Weight (kg) and height (m) were used to calculate BMI (body mass index). Body mass index (BMI) was calculated as body weight (kg) divided by height squared (m^2^). BMI values were compared with BMI norms for age and sex according to WHO criteria, thus obtaining a BMI z-score, which is a normalized relative weight indicator independent of age and sex.

### 2.3. Blood Sampling and Biochemical Analysis

For biochemical measurements, peripheral blood (3 mL) was taken in the morning after an overnight fast. Serum samples were obtained after centrifugation (2,500*g* at 4°C for 10 min) and were used for lipid profile, glucose, and C-reactive protein determination. Residual serum was stored in small portions at −70°C until analyses of TOC, TAC (max 4 weeks), and ox-LDL (max 6 months) were performed.

Total oxidant capacity (TOC) and total antioxidant capacity (TAC) values were measured by colorimetric assay (Labor Diagnostica Nord GmbH & Co. KG, Nordhorn, Germany). The method is based on the enzymatic reaction of peroxides and peroxidases. Oxygen produced by this reaction oxidizes the chromogenic substrate tetramethylbenzidine (TMB), which changes its colour from colourless to blue. By addition of sulfuric acid, the reaction cascade is stopped and the colour of the mixture changed to yellow and can be detected at 450 nm. Serum peroxide levels were calculated as the difference of the absorbance readings relating to the hydrogen peroxide standard curve. Antioxidants inhibit this reaction and can be detected analogously on the basis of the indirect proportionality of this inhibition reaction. The limit of detection was 0.06 mmol/L for TOC and 0.08 mmol/L for TAC. The intra- and interassay coefficients of variation (CV) were less than 4.9% and 7.33% for TOC and 2.5% and 3.33% for TAC, respectively. Oxidative stress index (OSI) was defined as the percentage ratio of TOC levels to TAC levels [21].

Oxidized-LDL (Ox-LDL) levels were determined by enzyme-linked immunosorbent assay (ELISA) (Immundiagnostik AG, Bensheim, Germany). The intra- and interassay coefficients of variability were found less than 5.7% and 9.0%, respectively. The detection limit was 4.13 ng/mL.

Total cholesterol (TC), HDL cholesterol (HDL-C), LDL cholesterol (LDL-C), and triglyceride (TG) concentrations as well as glucose and C-reactive protein (CRP) were determined by standard methods on Integra Cobas 400 plus analyzer (Roche Diagnostics, Basel, Switzerland).

### 2.4. Statistical Analysis

All of the statistical analyses were performed using Statistica 12 software. The Shapiro-Wilk test was used to evaluate the normality of variable distribution. The results are presented as means and standard deviations (SD) for normally distributed variables or medians and interquartile ranges for nonnormally distributed variables. Differences in baseline characteristics and biochemical parameters of obese and nonobese children were assessed using the Student *t*-test for normally distributed data and the Mann–Whitney *U* test for nonnormally distributed data. Pearson's correlation coefficients (*r*) were calculated to evaluate correlations between biochemical parameters, age, and BMI z-score was used for statistical analysis. A *p* value < 0.05 was considered statistically significant.

## 3. Results

Children in both studied groups did not differ in terms of age. The percentage of boys and girls was 35.5% (*n* = 22) and 64.5% (*n* = 40) in the obese group, and 47.6% (*n* = 10) and 52.4% (*n* = 11) in the nonobese group, respectively. BMI and BMI z-score were significantly higher (*p* < 0.001) in children with obesity compared with control peers. Average obesity duration was 3.5 (2.6–4.5) years. Characteristics of the studied children are presented in Table 1.

Serum TOC concentration was significantly (*p* < 0.05) higher, and TAC concentration was lower (*p* < 0.05) in children with obesity than in nonobese ones. The ox-LDL serum value was lower in children with obesity, but this difference was not statistically significant. Additionally, we observed significantly higher (*p* < 0.01) OSI in subjects with obesity compared with nonobese ones. Concentrations of glucose, total cholesterol, LDL cholesterol, and triglycerides did not differ statistically between both groups of children; however, serum HDL cholesterol levels were lower (*p* < 0.05) in children with obesity. CRP concentrations were low in all children, but the median value of this parameter was higher (*p* < 0.01) in the obese group. Serum concentrations of biochemical parameters in the studied children are presented in Table 2.

**Figure 1:**
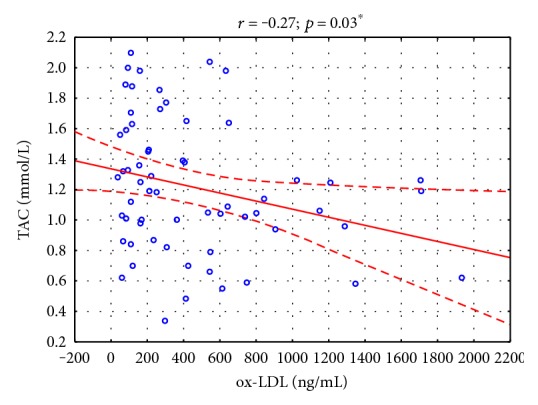
Correlations between serum TAC and ox-LDL concentrations in children with obesity (*N* = 62). ^∗^*p* < 0.05.

We found a significant negative correlation between serum TAC and ox-LDL concentrations (*r* = −0.27, *p* < 0.05) (Figure 1) and positive correlation between CRP and TAC concentrations (*r* = 0.36, *p* < 0.05) in children with obesity. We also observed that obesity duration was positively correlated with TOC level (*r* = 0.32, *p* < 0.05) in the obese group (Table 3).

## 4. Discussion

In people with obesity, the source of reactive oxygen species (ROS) responsible for oxidative stress could be the obesity itself and obesity-related accumulation of fat in the body as an independent factor of ROS formation, the generated chronic low-grade inflammatory state by obesity, and complications resulting from obesity [17, 18, 22].

Our study indicates that prepubertal children with obesity already have a greater intensification of oxidative processes measured by TOC concentrations and OSI values, while simultaneously lowered antioxidant defense measured by TAC concentrations compared with nonobese children. For total oxidant capacity determination, we used the method described by Tatzber et al. [23] based on oxidation of TMB by horseradish peroxidase (HRP)/H_2_O_2_. This type of reaction is the basic mechanism for detecting both peroxide levels and peroxidase activity in blood serum. In the case of measurement of total peroxides, HRP was added, while excess hydrogen peroxide was added for determination of enzyme activity [23]. Most previous studies on oxidative stress in children with obesity were conducted in children over 6 years old and adolescents. They provide evidence of the existence of a dependence between obesity and oxidative stress. Codoñer-Franch et al. [24] studying a group of children with obesity (6–14 years) with SDS-BMI ≥ 3 found significantly higher concentrations of free radical damage markers, such as malondialdehyde (MDA) and plasma carbonyl groups (CG) compared in nonobese children. Similarly, Albuali [25] showed that children with noncomplicated obesity aged 6–12 years with BMI > 35 kg/m^2^ had higher concentrations of lipid oxidation products, such as malonodialdehyde (MDA), ox-LDL, or advanced oxidation protein products (AOPPs), and lower concentrations of enzymes with antioxidative activities, such as superoxide dismutase (SOD), catalase (CAT), glutathione peroxidase (GSH-Px), glutathione reductase (GSSG-R), and glutathione (GSH), than the nonobese. Simultaneously, the authors found increased antioxidant enzyme activity in overweight children, which suggests that this may be a response to increased oxygen free radical production. This is concordant with previous findings, on the basis of which it was determined that in the early stages of obesity development, there is initially increased antioxidant enzyme activity, the aim of which is to prevent the effects of oxidative stress [26, 27].

This also pertains to TAC concentrations, which in the study of Kilic et al. [28] were significantly higher in the group of children with obesity compared with normal weight children. However, the authors demonstrated that increased TAC was the result of elevated TOC levels in an attempt to balance oxidation. They also suggest that the cause of greater TAC concentrations could be more active antioxidant systems in young people. However, reduced antioxidative activities in adults as well as children with obesity could be caused by considerable ROS production, which could be responsible for the depletion of antioxidant enzymes and a secondary reduction in the body's antioxidative capability. This seems to explain the observed increased as well as decreased TAC concentrations in people with obesity.

These discrepancies, according to Brown et al. [29], could be related to obesity duration. The positive correlation we found between obesity duration and TOC concentration may confirm the intensification of oxidative processes along with the duration of obesity occurrence not only in adults but also in children. Taking into account that our study included children up to 10 years old, with a relatively short obesity duration—median obesity duration in this group of children was 3.5 (2.6–4.5) years—one should expect elevated TAC levels in accordance with the concept of Brown et al. as well as other authors' observations. All the more so that CRP concentration as a marker of low-grade inflammation was significantly higher in the group of children with obesity and HDL cholesterol, a fraction that shows anti-inflammatory and antioxidative activity was lower in this group; however, both parameters were in the reference value range. Furthermore, CRP showed a positive correlation with TAC, and ox-LDL concentrations were similar in both groups. All this could mean the onset of an elevated response of antioxidant mechanisms of the body to oxidative stress.

We observed not only lower TAC concentrations in children with obesity but also elevated OSI levels compared with nonobese ones. Additionally, TAC exhibited a negative correlation with ox-LDL. This is all the more interesting because the studied children with obesity did not have markers of metabolic syndrome, which would explain not only the increased TOC but also decreased TAC and higher OSI in such small children.

Vehapoglu et al. [30], in a study of similarly-aged children (2–11 years) to our studied groups, also found significantly lowered TAC concentrations but did not demonstrate a difference in TOC or OSI levels in children with obesity compared with children of normal weight or underweight. On the other hand, Eren et al. [31] found that increased TOC and OSI levels can be accompanied by elevated TAC concentration in children with obesity with metabolic syndrome compared with children with obesity without metabolic syndrome and children of normal weight. With so many different observations and dependencies established between oxidative stress markers and BMI, it can be assumed that excess adipose tissue is the main factor responsible for increased oxidative stress [32]. This is supported by an intervention study (a 4-week diet and exercise regimen) conducted among teenagers with obesity, which showed that a reduction in body mass positively affected oxidative stress parameters [33]. This claim could also be supported by the observation of Santoro et al. who demonstrated that in obese adolescents, free oxygen radicals are responsible for oxidation of not only LDL but also fatty acids derived from the linoleic acid (LA). This results in generation of oxidized derivatives of LA (oxidized LA metabolites: OXLAMs), which are deleterious for the hepatocyte and may cause liver injury, while the diet with a lower intake of *n* = 6 PUFAs may help reduce the damage, as observed, for example, in people suffering from NAFLD (nonalcoholic fatty liver disease) [34].

Although obesity increases oxidative stress even during childhood, it is difficult to identify a typical oxidative stress-related response, because most studies include different oxidative stress markers. ROS are produced during metabolic reactions, but it is largely unknown which factors modulate their production [22].

Due to the fact that our study is one of a few concerning prepubertal children, we are treating it as a pilot investigation. It is possible that including a larger group of younger children with obesity or use of other methods to assess oxidative stress would provide more important information on the oxidant and antioxidant status in this group.

The present study had several limitations. First, our sample size was relatively small, particularly the control group. However, both studied groups were similar in age, which may be an important factor for oxidative stress marker levels. Second, both groups differed in terms of sex distribution. There is, however, no unequivocal evidence that sex affects the intensification of oxidative stress in children with obesity. Although Sobieska et al. [35] suggest that girls are more resistant to the negative effects of increased adiposity status compared with boys, Kilic et al. [28] did not confirm such a dependence. It should also be taken into account that these observations concerned children over 6 years old and teenagers, while all children included in our study were prepubertal.

In summary, obesity-related oxidative stress already occurs in early childhood; elevated TOC and OSI levels as well as lowered TAC in the blood serum of prepubertal children may be evidence of this. Early obesity diagnosis and the necessary therapeutic activity implementation is a vital strategy for the prophylaxis of free radical damage and related multiorgan complications.

## Figures and Tables

**Table 1:** Characteristics of the studied children.

Variable	Obese children (*n* = 62)	Nonobese children (*n* = 21)	*p* value
Age (years)^+^	7.5 (6.3–8.8)	6.4 (5.5–8.6)	0.130
Weight (kg)^++^	40.2 ± 9.3	22.2 ± 6.6	<0.001^∗^
Height (cm)^+^	130.5 (121.0–141.0)	116.0 (102.5–129.0)	0.001^∗^
BMI (kg/m^2^)^+^	23.5 (21.9–24.6)	15.5 (15.2–16.3)	<0.001^∗^
BMI z-score^+^	3.0 (2.5–3.5)	−0.03 (−0.5–0.7)	<0.001^∗^

^+^Data are presented as median value and interquartile ranges (1Q–3Q); ^++^data are presented as mean value and standard deviation (SD); ^∗^*p* < 0.05; BMI: body mass index; BMI z-score: a normalized relative weight indicator independent of age and sex.

**Table 2:** Biochemical parameters in obese and nonobese children.

Variable	Obese children (*n* = 62)	Nonobese children (*n* = 21)	*p* value
TOC (mmol/L)^+^	0.22 (0.15–0.28)	0.15 (0.14–0.23)	0.03^∗^
TAC (mmol/L)^++^	1.22 ± 0.44	1.47 ± 0.52	0.03^∗^
Ox-LDL (ng/mL)^+^	283.8 (114.9–633.9)	363.8 (133.9–611.6)	0.60
OSI^+^	0.18 (0.12–0.27)	0.11 (0.08–0.18)	0.006^∗^
CRP (mg/L)^+^	0.66 (0.43–1.66)	0.24 (0.10–0.59)	0.008^∗^
Glucose (mg/dL)^+^	86.0 (83.0–90.0)	91.0 (83.0–92.0)	0.50
Cholesterol total (mg/dL)^+^	164.0 (150.0–194.5)	164.5 (157.0–186.0)	0.90
Cholesterol HDL (mg/dL)^+^	51.0 (43.5–59.5)	63.0 (58.0–73.0)	0.04^∗^
Cholesterol LDL (mg/dL)^+^	109.0 (94.0–129.0)	116.0 (88.0–120.0)	0.90
Triglycerides (mg/dL)^+^	72.5 (58.5–86.0)	55.0 (48.0–91.0)	0.40

^+^Data are presented as median value and interquartile ranges (1Q–3Q); ^++^data are presented as mean value and standard deviation (SD); ^∗^*p* < 0.05; TOC: total oxidant capacity; TAC: total antioxidant capacity; ox-LDL: oxidized low-density lipoprotein; OSI: oxidative stress index; CRP: C-reactive protein.

**Table 3:** Correlations between serum concentrations of oxidative status markers and clinical/biochemical parameters in children with obesity (*N* = 62).

	TOC		TAC		OSI		ox-LDL	
	*r*	*p* value	*r*	*p* value	*r*	*p* value	*r*	*p* value
Duration of obesity	0.32	0.01^∗^	0.15	0.2	0.17	0.2	−0.16	0.2
Age	0.12	0.4	0.09	0.5	0.03	0.8	−0.09	0.5
BMI z-score	0.03	0.8	0.15	0.2	−0.09	0.4	−0.15	0.2
CRP	0.20	0.2	0.36	0.009^∗^	−0.04	0.8	0.05	0.7
Glucose	0.06	0.7	0.10	0.5	0.06	0.6	−0.14	0.3
Cholesterol total	−0.08	0.6	−0.06	0.6	−0.01	0.9	0.01	0.9
HDL cholesterol	0.01	0.9	−0.04	0.7	−0.04	0.8	0.10	0.5
LDL cholesterol	−0.12	0.4	−0.03	0.8	−0.06	0.7	0.01	0.9
Triglycerides	−0.03	0.8	−0.07	0.6	0.09	0.5	0.07	0.6

*r*: Pearson's correlation coefficient; ^∗^*p* < 0.05; TOC: total oxidant capacity; TAC: total antioxidant capacity; ox-LDL: oxidized low-density lipoprotein; OSI: oxidative stress index; CRP: C-reactive protein.
